# Supplementary material for: Harnessing enzyme promiscuity of alditol-2-dehydrogenases for oxidation of alditols to enantiopure ketoses
Source: PLoS One. 2025 Jun 25;20(6):e0325955. doi: 10.1371/journal.pone.0325955 (PMC12193009; doi:10.1371/journal.pone.0325955)
Supplement: S1 Fig — (DOCX) [file pone.0325955.s001.docx]

**Supporting Information**

**S1 Fig.**

**Harnessing Enzyme Promiscuity of Alditol-2-Dehydrogenases for Oxidation of Alditols to Enantiopure Ketoses**


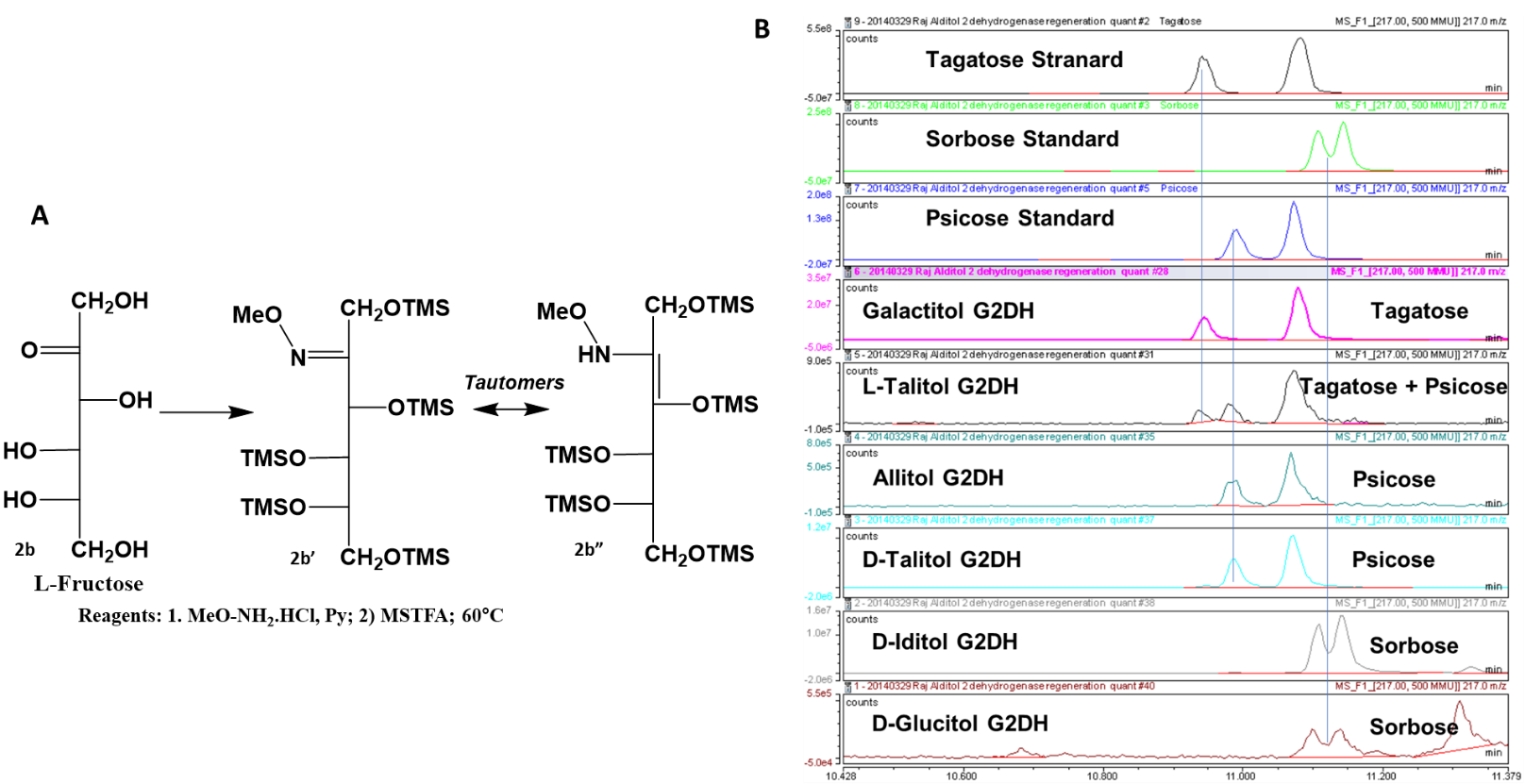


**Figure S1A:** **A: Methoxime-TMS Derivatization scheme for GC/MS detection of ketoses; tautomers in equilibrium (L-fructose (2b) is an example). B: Detection of product ketoses from alditol G2DH reactions after methyloxime-TMS derivatization^29^ by GCMS examples:** Top three panels are tagatose (**3a,b**), sorbose (**4a,b**) and psicose (**1a,b**) respectively; tagatose produced from galactitol (**5a,b**)(4th), mix of tagatose (**3b**) and psicose (**1b**) from L-talitol (**8b**)(5th), psicose (**1b**) from allitol (**10a,b**)(6^th^) and D-talitol (**3b**) (7^th^), sorbose (**4a**) from D-iditol (**12a**)(8^th^) and D-glucitol (**9a**)(9^th^).


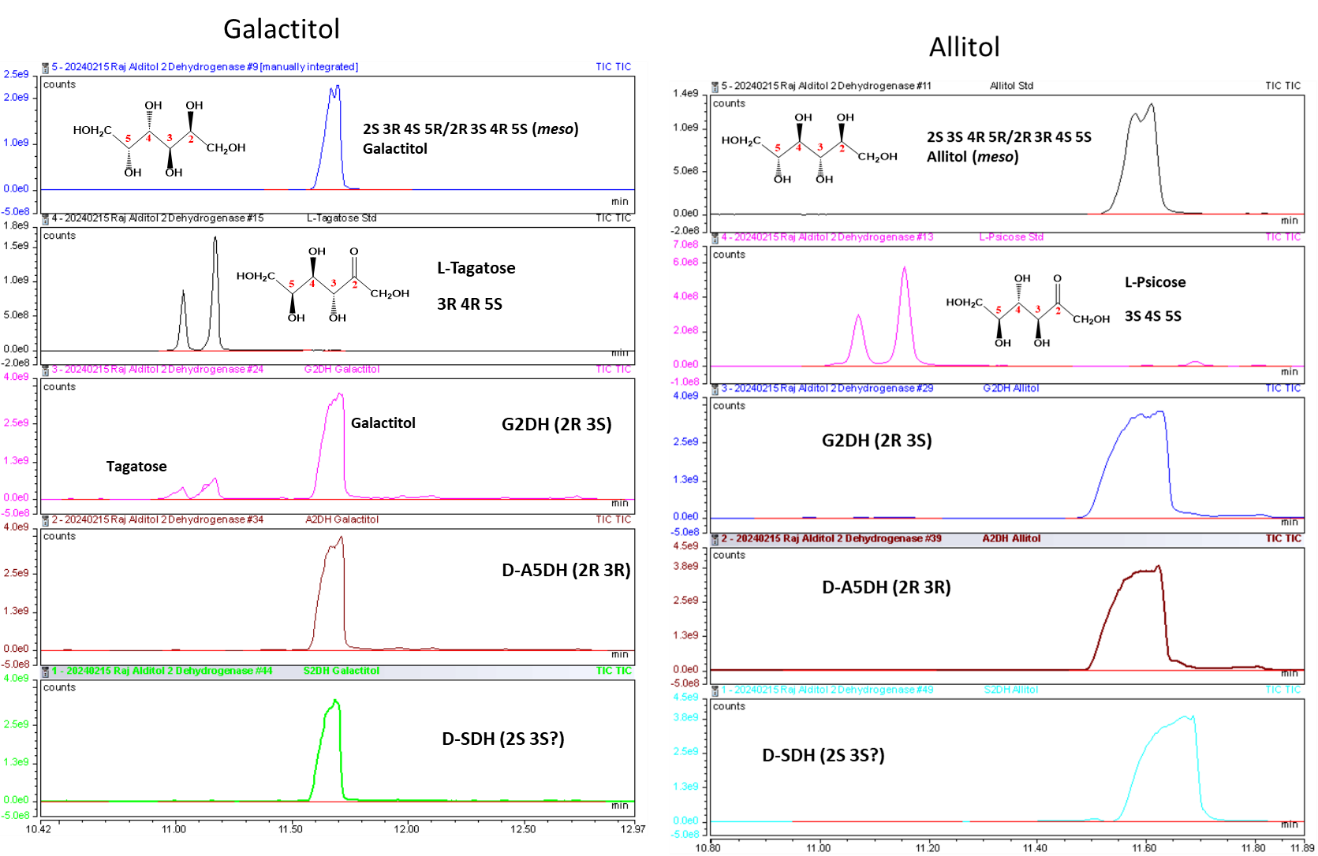


**Fig S1B: Chromatograms for** **reaction of Galactitol and Allitol with three enzymes**; GC/MS detection of ketoses using Methoxime-TMS method; Substrate aditol (Top panel, galactitol (Left); Allitol (Right)), Reference Product ketose (2^nd^ panel, tagatose (Left); Psicose (Right)). Reaction with G2DH (3^rd^ panel, tagatose (left) and weak psicose (right) (see **Fig 3.** For clear detection), Reaction with D-A5DH (4^th^ panel) and reaction with D-S2DH (5^th^ panel).


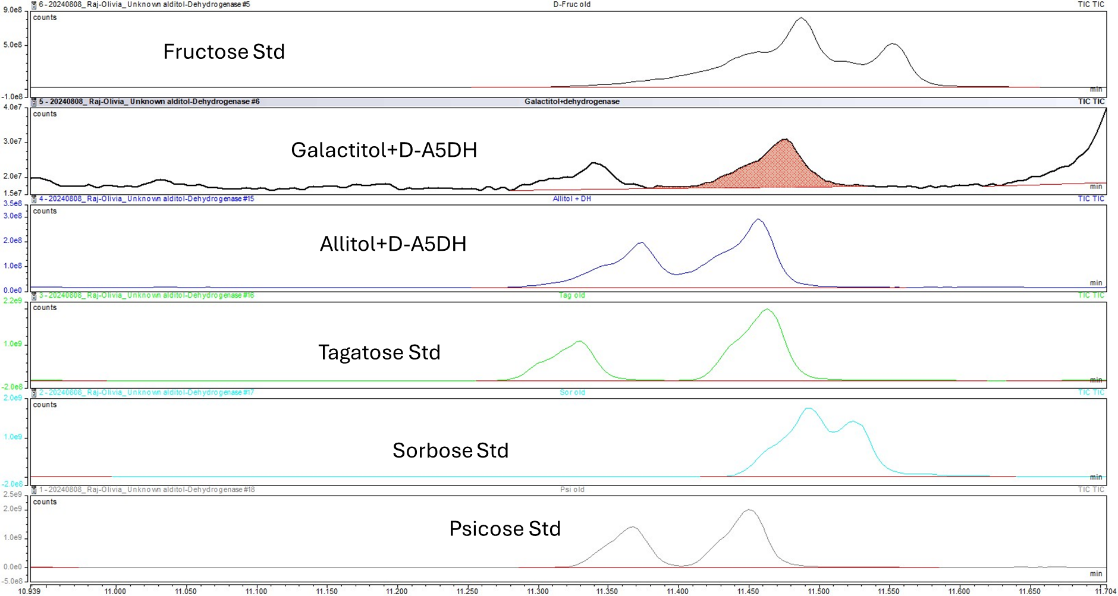


**Fig S1C: Chromatograms for Chromatograms for** **reaction of galactitol and allitol with D-A5DH.** Products of D-A5DH reaction with *meso* alditol (galactitol (2^nd^ panel) and allitol (3^rd^ panel)). Galactitol produced tagatose and Allitol produced psicose.


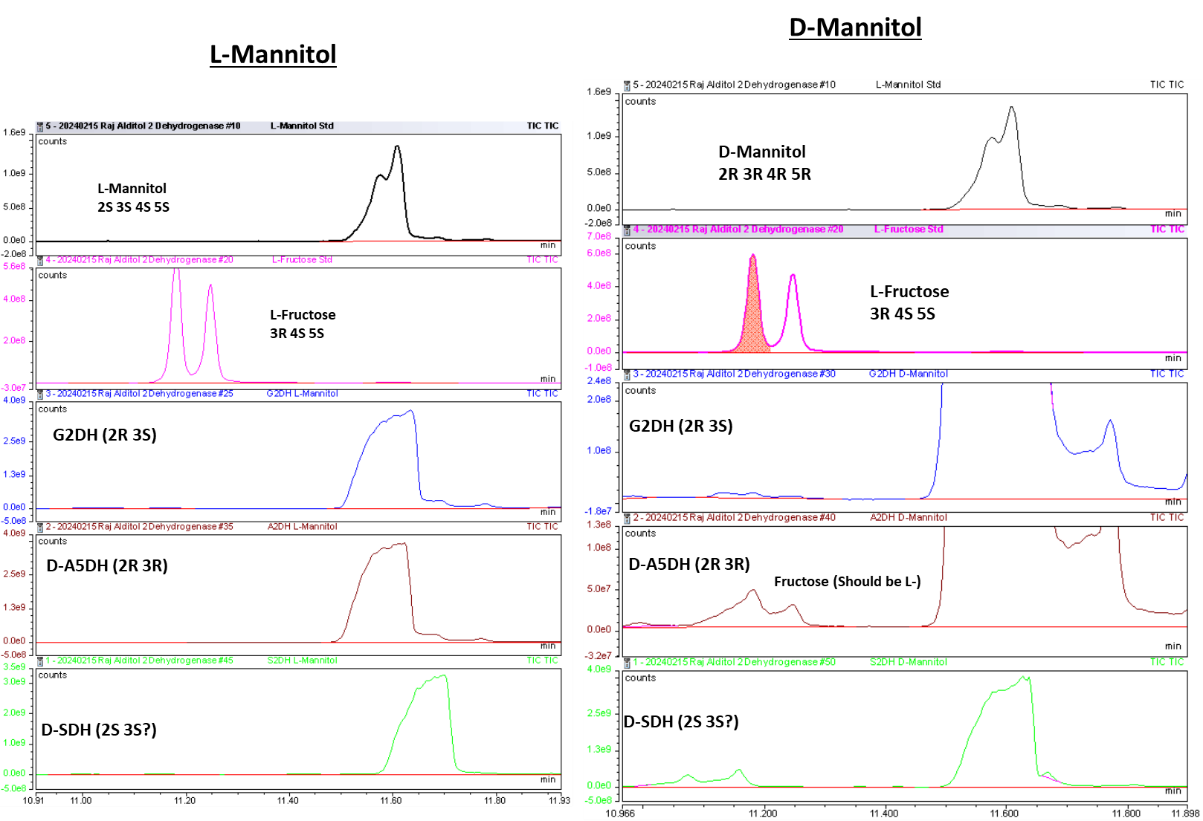


**Fig S1D:** **Chromatograms for** **reaction of D-/L-mannitol with three enzymes**. GC/MS detection of ketoses using Methoxime-TMS method; Substrate aditol (Top panel, L-mannitol (Left); D-mannitol (Right)), Reference Product ketose ( 2^nd^ panel, fructose). Reaction with G2DH (3^rd^ panel), Reaction with D-A5DH (4^th^ panel; product fructose (right)) and reaction with D-S2DH (5^th^ panel).


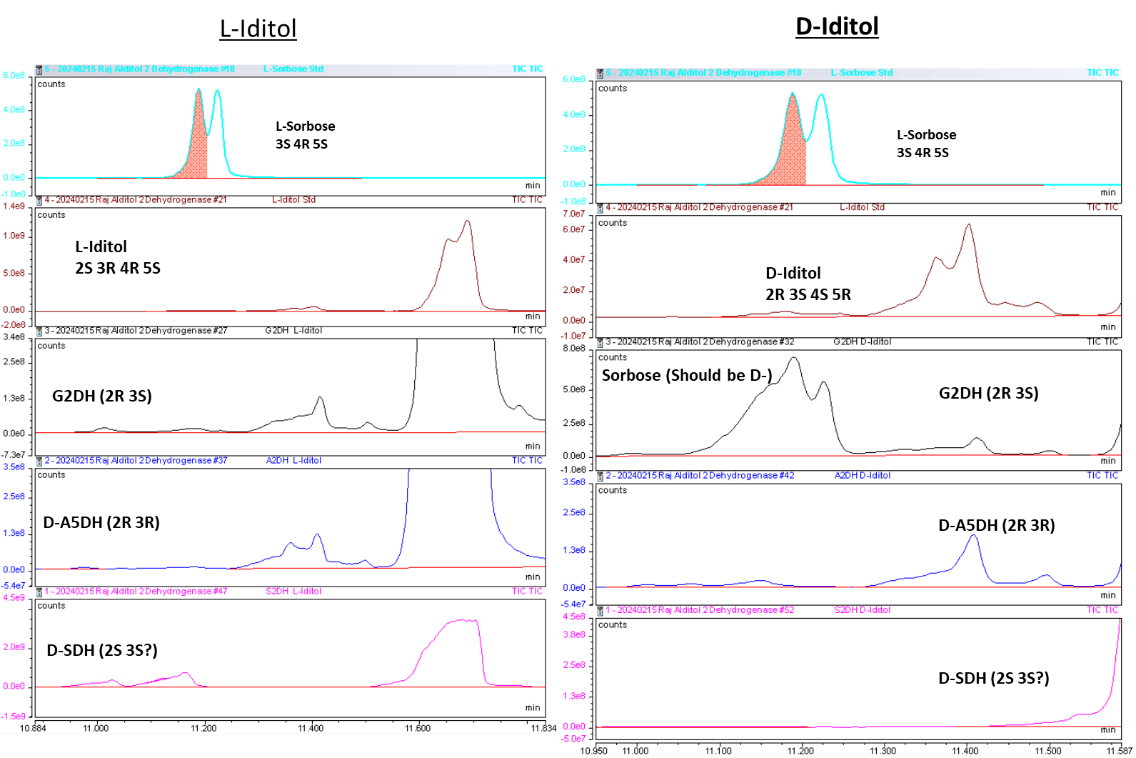


**Fig S1E:** **Chromatograms for** **reaction of D-/L-iditol with three enzymes.** GC/MS detection of ketoses using Methoxime-TMS method; Reference Product ketose (top panel, sorbose, Substrate aditol (2^nd^ panel panel, L-iditol (Left); D-iditol (Right)). Reaction with G2DH (3^rd^ panel, product sorbose (right)), Reaction with D-A5DH (4^th^ panel) and reaction with D-S2DH (5^th^ panel).


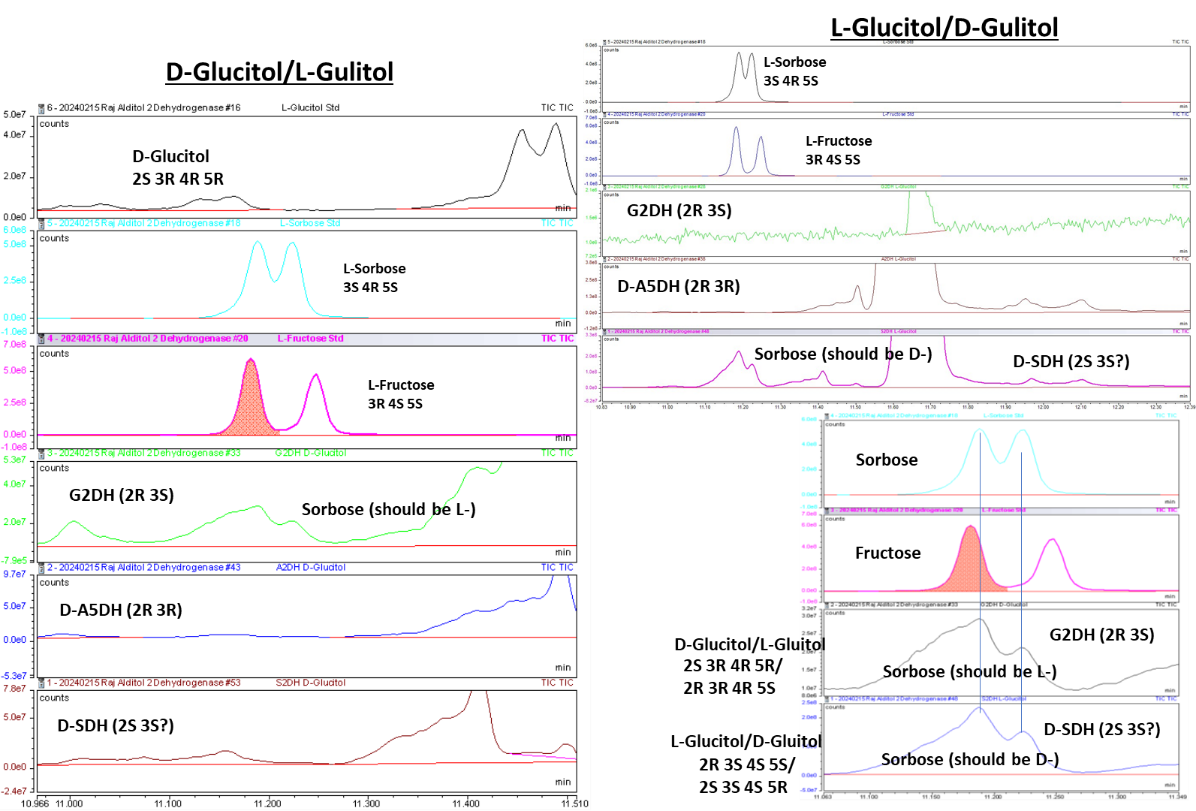


**Fig S1F:** **Chromatograms for** **reaction of D-L-glucitol/L-/D-gulitol with three enzymes.** GC/MS detection of ketoses using Methoxime-TMS method; **Left:** Substrate aditol (Top panel, D-glucitol/L-gulitol (Left), Reference Product ketoses (2^nd^ panel, sorbose, 3^rd^ panel fructose). Reaction with G2DH (4th panel, sorbose, Reaction with D-A5DH (4^th^ panel) and reaction with D-S2DH (5^th^ panel). ); **Right**: L-glucitol/D-gulitol (Right)) Reference Product ketoses (1^st^ panel sorbose, and 2^nd^ panel fructose) Reaction with G2DH (3rd panel, Reaction with D-A5DH (4^th^ panel) and reaction with D-S2DH (5^th^ panel, Sorbose product); Bottom 4 panels confirming retention times of sorbose as products.


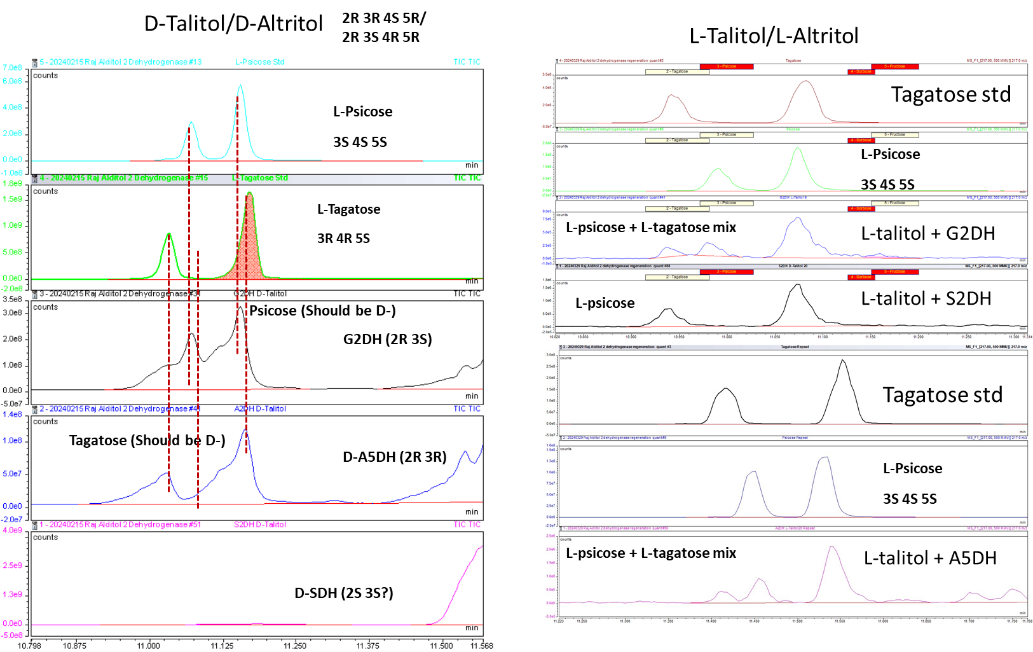


**Fig S1G:** **Chromatograms for** **reaction of D-altritol/talitol and L-altritol/talitol with three enzymes.** GC/MS detection of ketoses using Methoxime-TMS method; **Left: For D-talitol/D-altritol**, Reference product standards psicose (top panel) tagatose (2^nd^), mix of psicose and tagatose products with G2DH (3^rd^), product tagatose with D-A5DH (4^th^) and no product with D-S2DH (5^th^). **Right:** **for L-talitol/L-altritol**, Reference product standards tagatose (top panel and 5^th^), psicose (2^nd^ and 6^th^), mix of psicose and tagatose products with G2DH (3^rd^), product tagatose with D-S2DH (4^th^) and mix of psicose and tagatose products with D-A5DH (7^th^).
