# Supplementary material for: Harnessing enzyme promiscuity of alditol-2-dehydrogenases for oxidation of alditols to enantiopure ketoses
Source: PLoS One. 2025 Jun 25;20(6):e0325955. doi: 10.1371/journal.pone.0325955 (PMC12193009; doi:10.1371/journal.pone.0325955)
Supplement: S2 Fig — The linear plot of Ketose/xylulose (internal standard) GC/MS intensity ratio vs concentration. LOD was calculated from regression analysis. (DOCX) [file pone.0325955.s002.docx]

**Supporting Information**

**S2 Fig.**

**Harnessing Enzyme Promiscuity of Alditol-2-Dehydrogenases for Oxidation of Alditols to Enantiopure Ketoses**


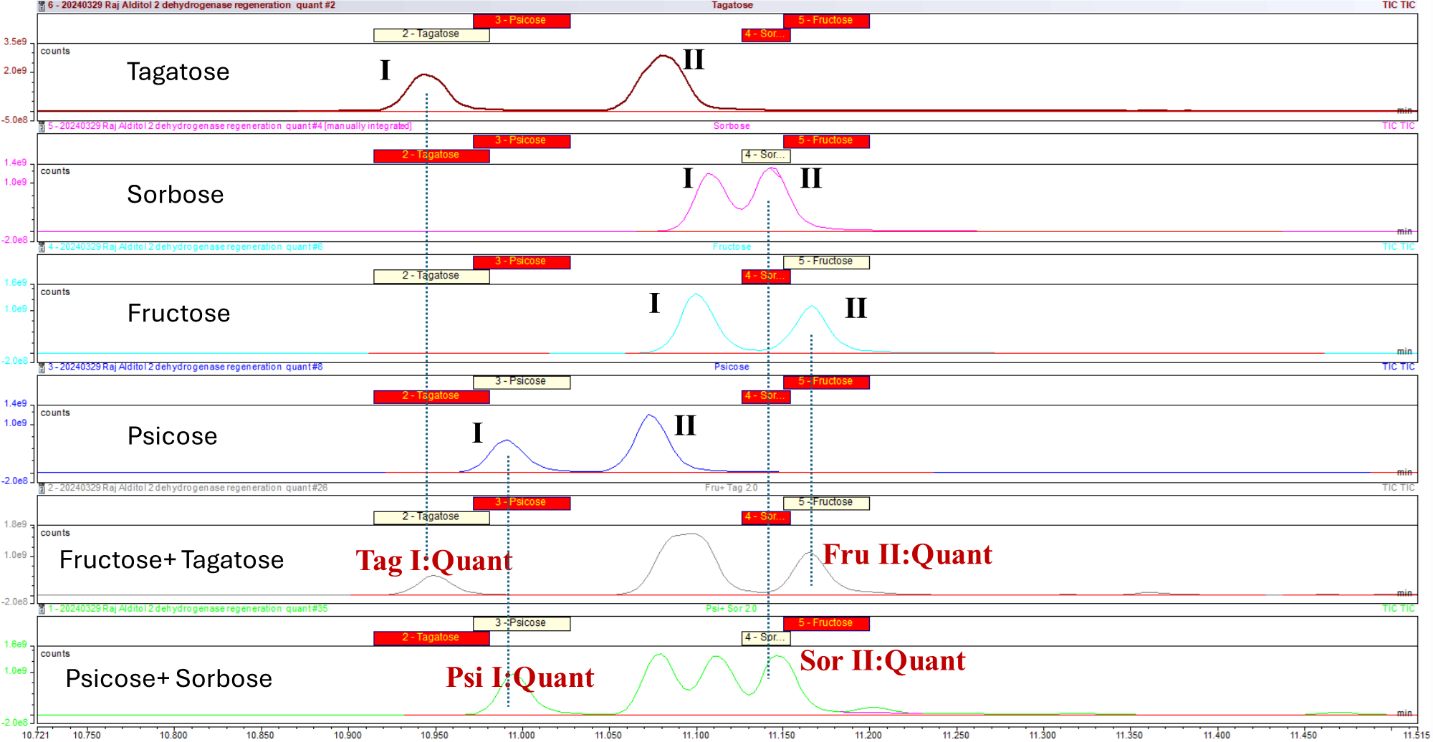


**Fig S2A:** GC/MS chromatogram explaining peaks assigned for quantitation of ketoses


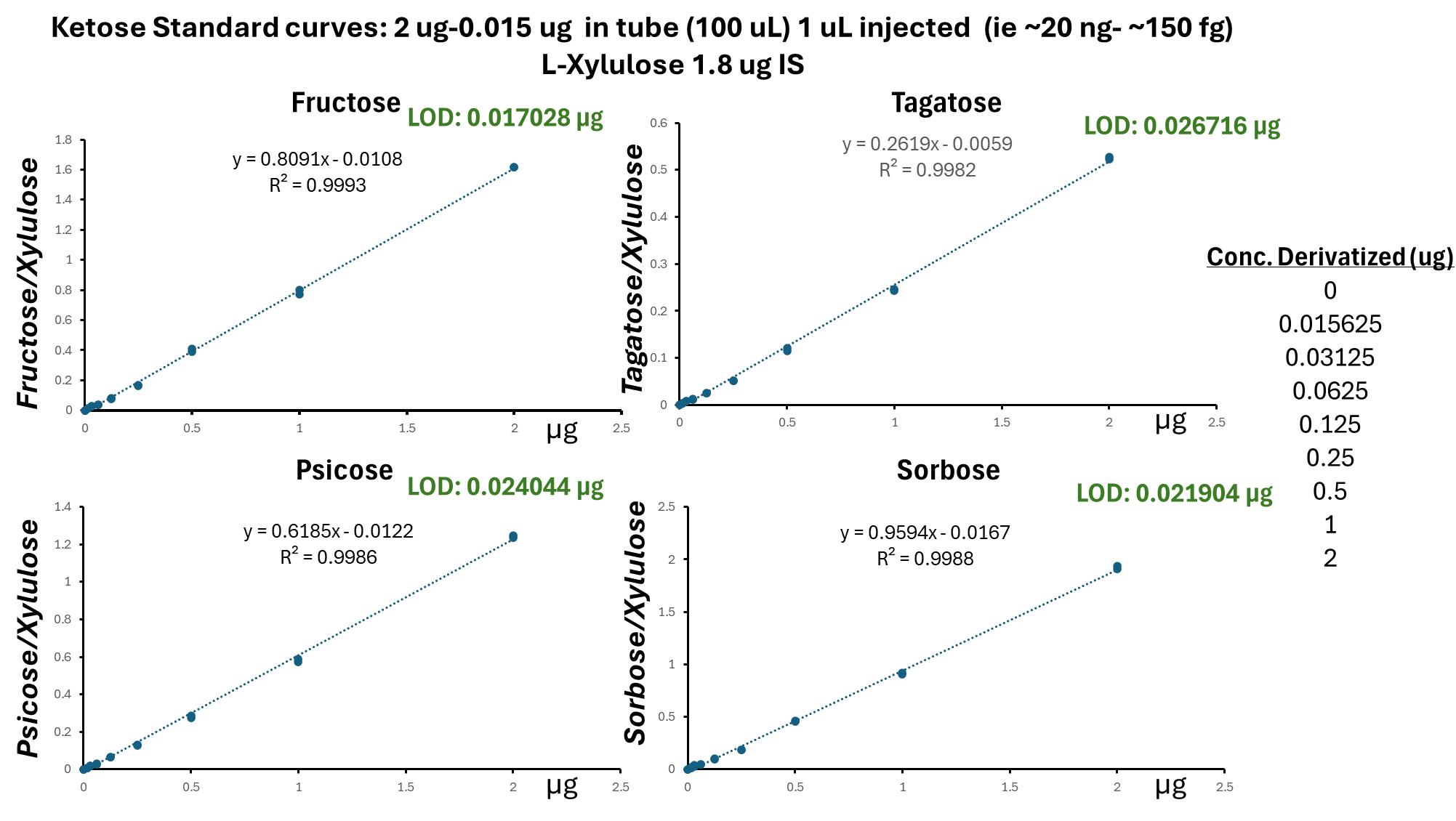


**Fig S2B:** The linear plot of Ketose/xylulose (internal standard) GC/MS intensity ratio *vs* concentration. LOD was calculated from regression analysis. Detailed calculation presented in Supporting Information S3 Dataset (Excel file)
