# Supplementary material for: Harnessing enzyme promiscuity of alditol-2-dehydrogenases for oxidation of alditols to enantiopure ketoses
Source: PLoS One. 2025 Jun 25;20(6):e0325955. doi: 10.1371/journal.pone.0325955 (PMC12193009; doi:10.1371/journal.pone.0325955)
Supplement: S4 Fig — GC/MS characterization of enantiopure ketose products from alditols with G2DH/D-A5DH/D-S2DH reactions. (DOCX) [file pone.0325955.s004.docx]

**Supporting Information**

**S4 Fig.**

**Harnessing Enzyme Promiscuity of Alditol-2-Dehydrogenases for Oxidation of Alditols to Enantiopure Ketoses**

**CHIRAL GC/MS**


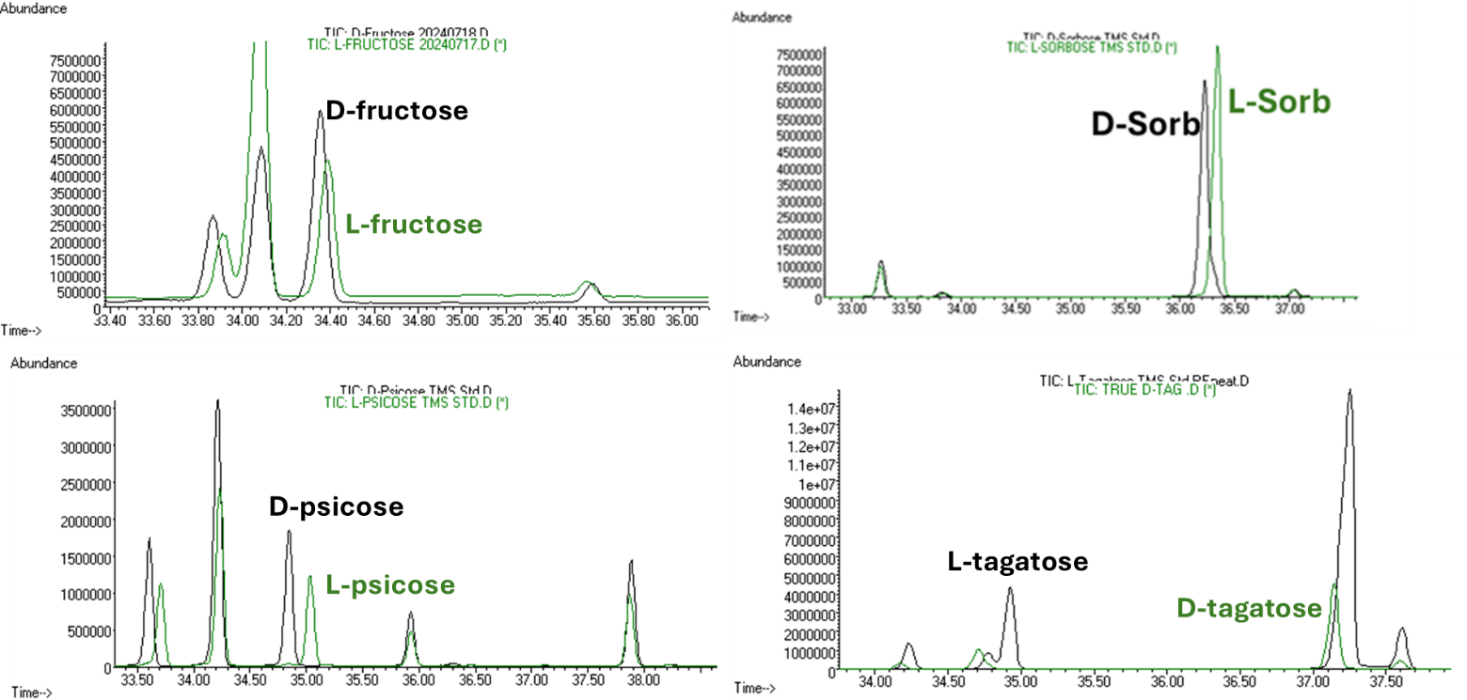


**Fig S4A:** GC/MS Chromatograms of standard Ketose enantiomers derivatized with trimethylsilyl groups locking conformations as diastereomers and run using CP-Chirasil-Dex CB (Agilent CP7502) column. **Top Left:** D-fructose(black), L-fructose (green); **Top Right:** D-sorbose(black), L-fsorbose (green); **Bottom Left**: D-psicose(black), L-psicose (green); **Bottom Right:** D-tagatose (green), L-tagatose (black).

**Chiral G2DH**


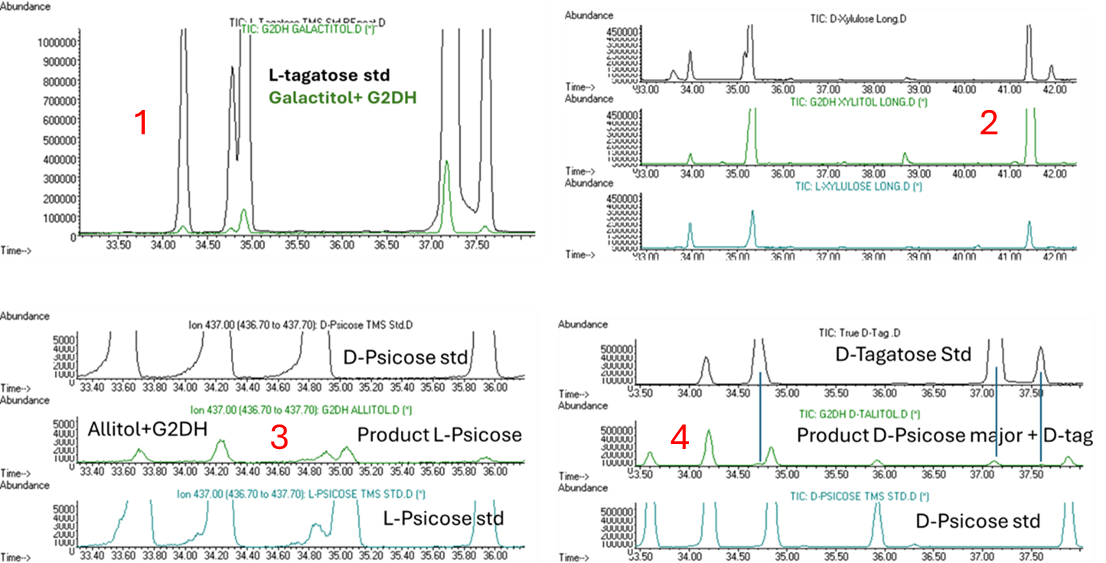


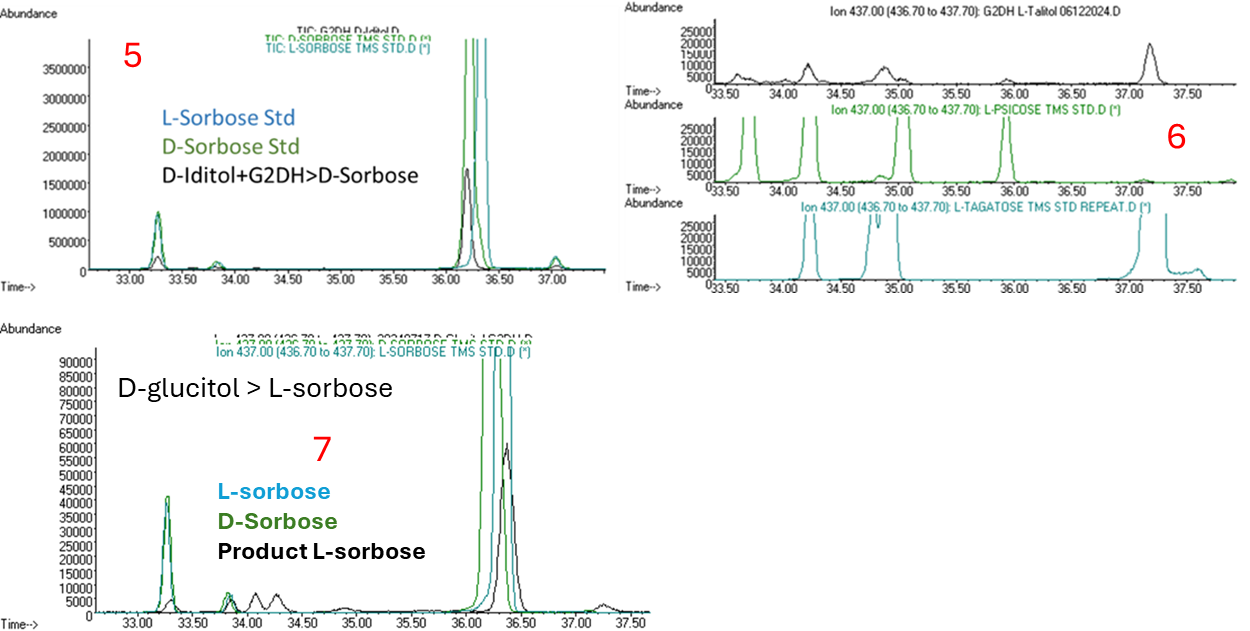


**Fig S4B:** GC/MS characterization of enantiopure ketose products from alditols and **G2DH** reactions. **1**. L-tagatose from galactitol, **2**, L-xylulose from xylitol, **3**, L-psicose from allitol, **4**. D-psicose from D-talitol (D-tagatose, the minor product not visible), **5**. D-sorbose from D-iditol, **6**. mixture of L-tagatose and L-psicose from L-talitol.

**Chiral D-A5DH**


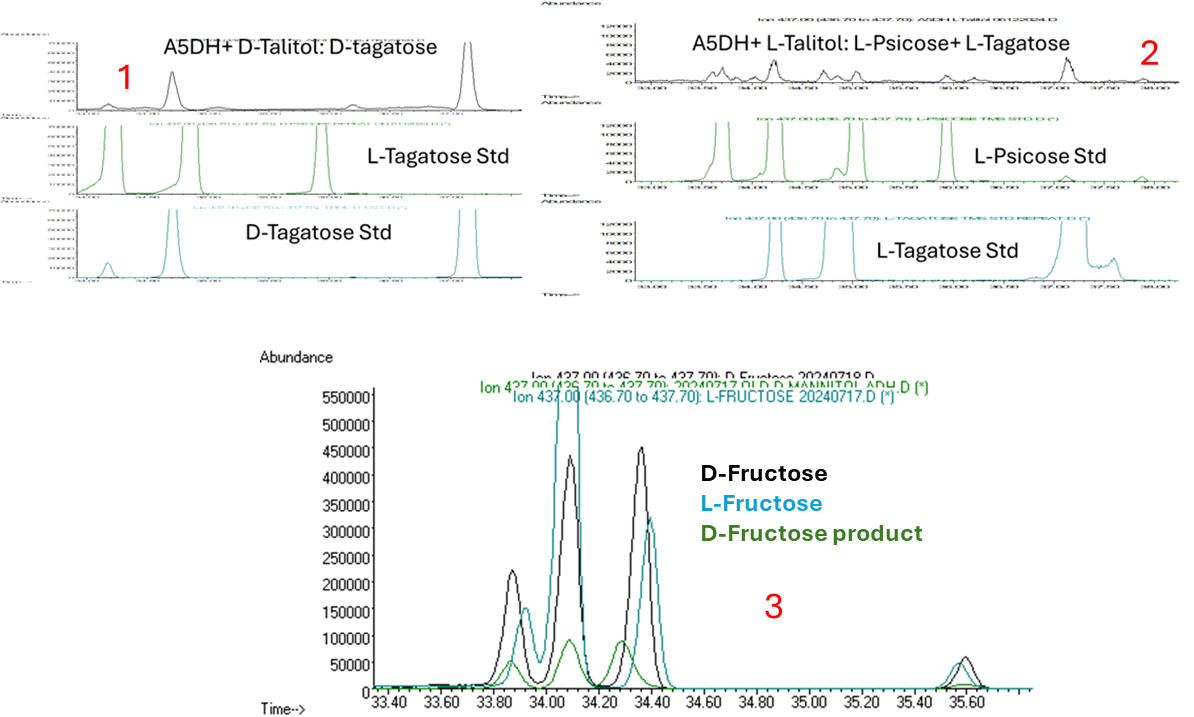


**Fig S4C:** GC/MS characterization of enantiopure ketose products from alditols and **D-A5DH** reactions. 1. D-tagatose from D-talitol, 2. Mixture of L-tagatose and L-psicose from L-talitol, 3. D-fructose from D-mannitol.

**Chiral D-S2DH**


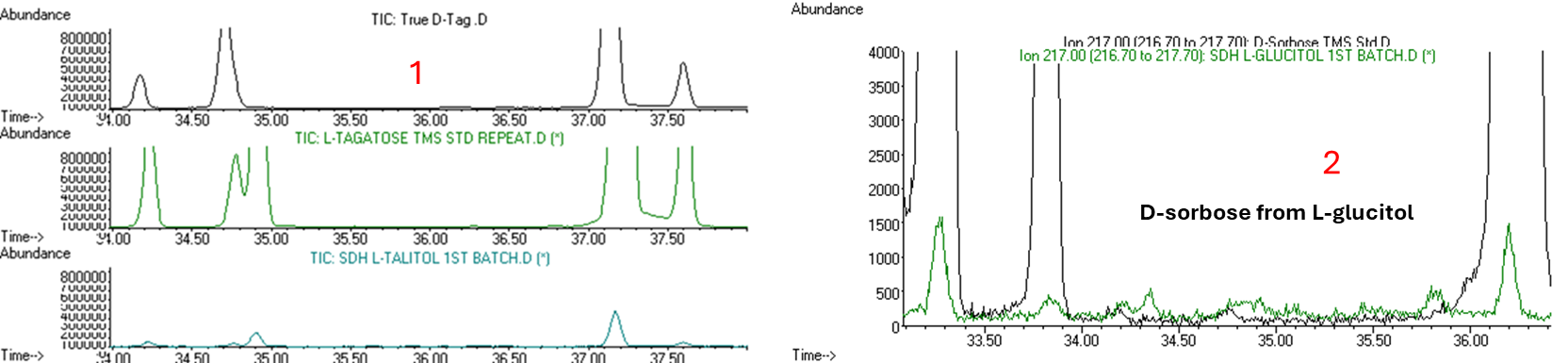


**Fig S4D:** GC/MS characterization of enantiopure ketose products from alditols and **D-S2DH** reactions. 1. Ltagatose from L-talitol, 2. D-sorbose from L-glucitol.
