# Supplementary material for: Harnessing enzyme promiscuity of alditol-2-dehydrogenases for oxidation of alditols to enantiopure ketoses
Source: PLoS One. 2025 Jun 25;20(6):e0325955. doi: 10.1371/journal.pone.0325955 (PMC12193009; doi:10.1371/journal.pone.0325955)
Supplement: S5 Fig — (DOCX) [file pone.0325955.s005.docx]

**Supporting Information**

**S5 Fig.**

**Harnessing Enzyme Promiscuity of Alditol-2-Dehydrogenases for Oxidation of Alditols to Enantiopure Ketoses**


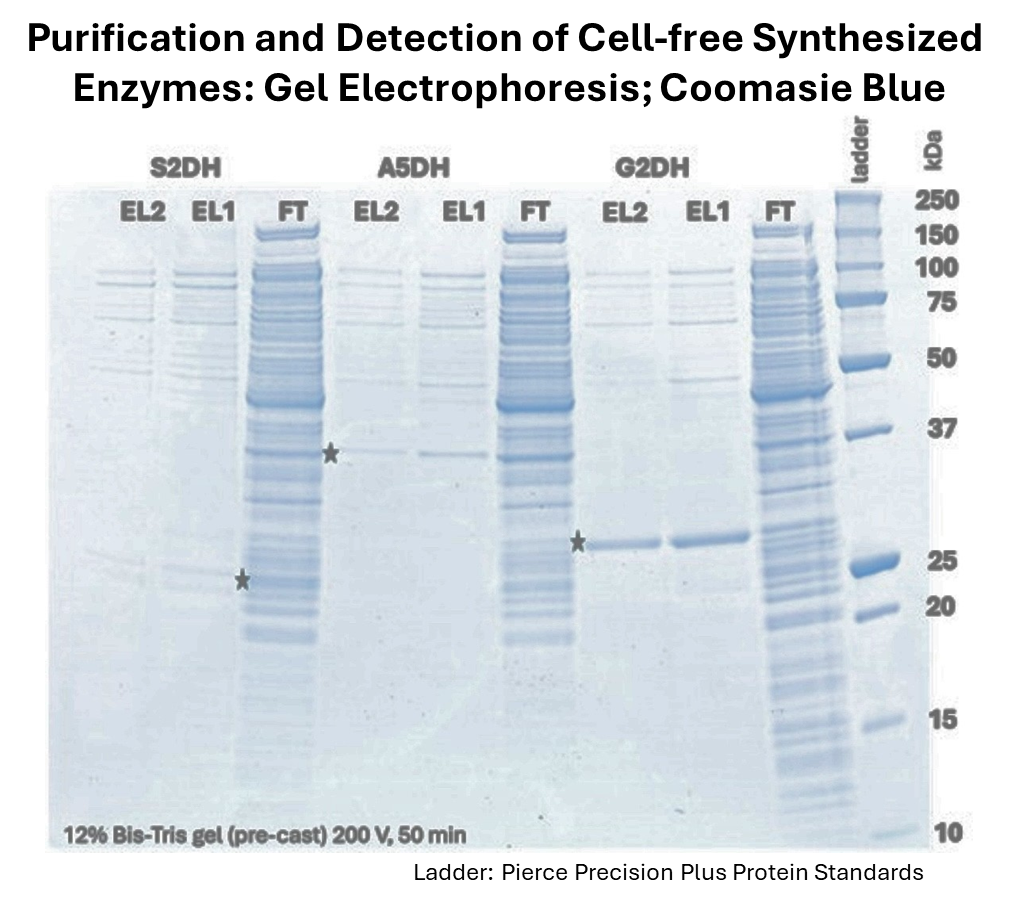


**Fig S5: Purification of cell-free synthesized enzymes:** Size confirmation for each protein was performed by SDS-PAGE run at 200V for 50 minutes using NuPage 12% Bis-Tris Mini-Gels (Invitrogen) and Precision Plus Protein Unstained Standards (Biorad) ladder, followed by visualization with InstantBlue Coomassie Protein Stain (Abcam).
