# Supplementary material for: Harnessing enzyme promiscuity of alditol-2-dehydrogenases for oxidation of alditols to enantiopure ketoses
Source: PLoS One. 2025 Jun 25;20(6):e0325955. doi: 10.1371/journal.pone.0325955 (PMC12193009; doi:10.1371/journal.pone.0325955)
Supplement: S6 Fig — (DOCX) [file pone.0325955.s006.docx]

**Supporting Information**

**S6 Fig.**

**Harnessing Enzyme Promiscuity of Alditol-2-Dehydrogenases for Oxidation of Alditols to Enantiopure Ketoses**


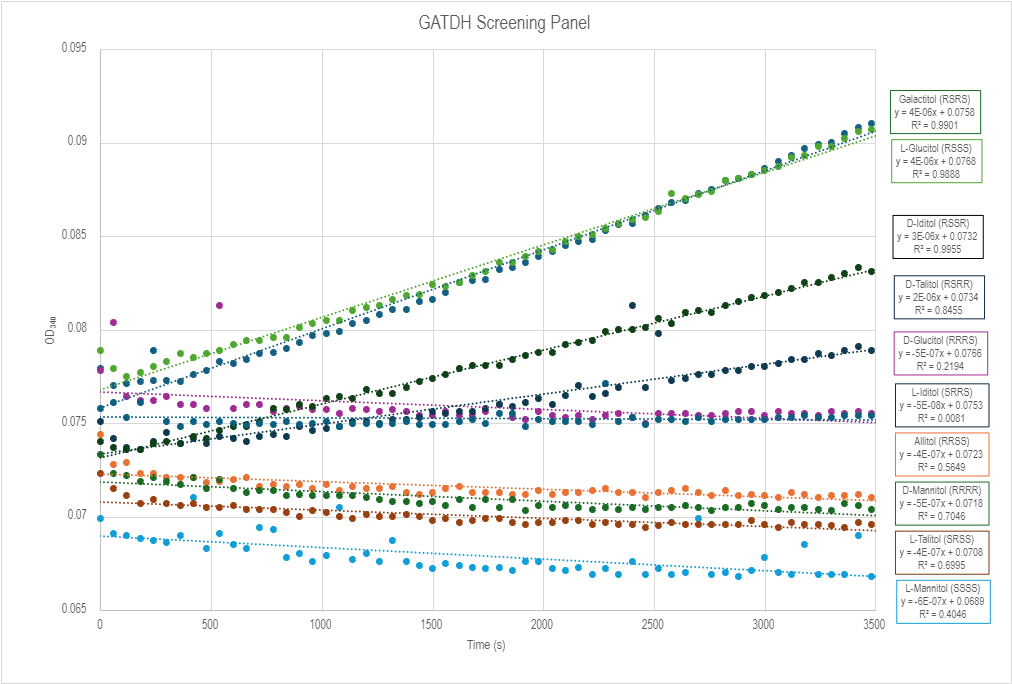


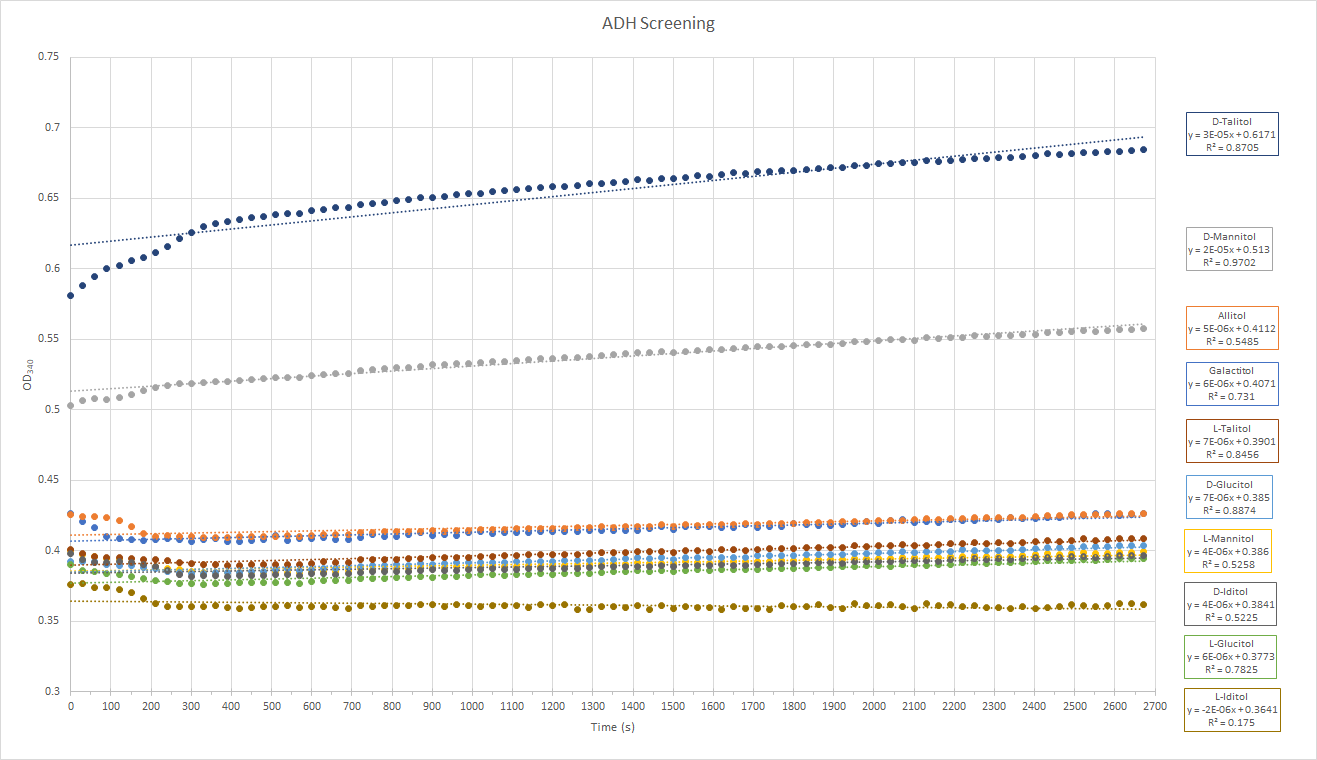


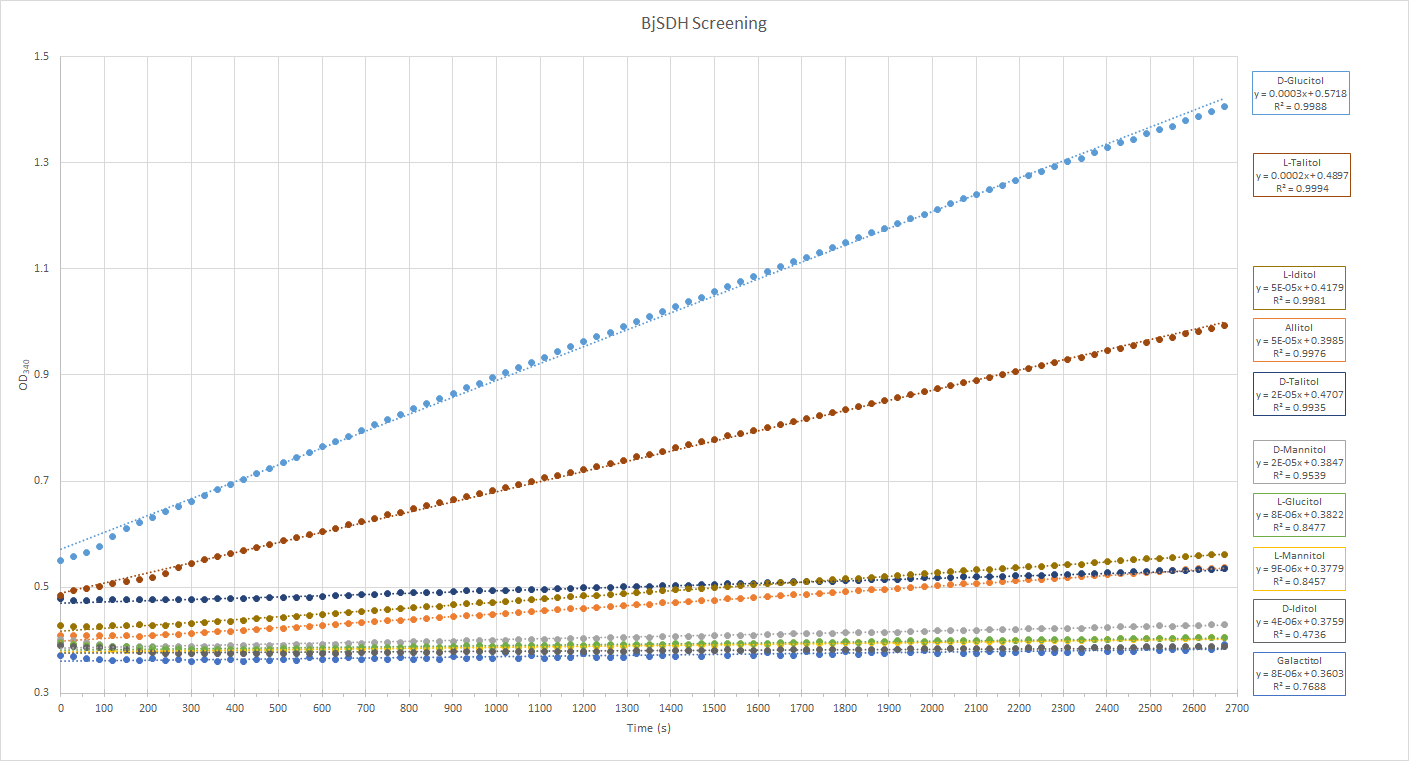


**Fig S6:** The initial reaction progress was monitored by measuring the absorbance (340 nM) of NADH using a TECAN Spark at a controlled temperature of 30°C until the period at which no further increment in NADH was observed For G2DH (Top) D-A5DH (2^nd^) and D-S2DH (3^rd^ ).
