# Supplementary material for: Harnessing enzyme promiscuity of alditol-2-dehydrogenases for oxidation of alditols to enantiopure ketoses
Source: PLoS One. 2025 Jun 25;20(6):e0325955. doi: 10.1371/journal.pone.0325955 (PMC12193009; doi:10.1371/journal.pone.0325955)
Supplement: S7 Fig — (DOCX) [file pone.0325955.s007.docx]

**Supporting Information**

**S7 Fig.**

**Harnessing Enzyme Promiscuity of Alditol-2-Dehydrogenases for Oxidation of Alditols to Enantiopure Ketoses**

**Isolation of ketose product from G2DH oxidation of 2-13C-D-galactitol**

The ketose product of 2-^13^C-D-galactitol oxidation was isolated from the reaction mixture using a Waters Semi Preparative HPLC-PDA instrument. The reaction components were separated using a CarboSep CHO-620 analytical column and a CarboSep CHO-87C guard column. The mobile phase is comprised of HPLC grade water at a flow rate of 0.5 mL min^-1^. Retention times for the ketose products were determined from the elution of 3 unlabeled ketose standard L-tagatose. Unlabeled galactitol (from TCI Chemicals) was also used as standards to identify the retention time of the alditol substrates. 1.5 mg of product and substrate standard was dissolved in HPLC grade water and 20 μL of each sample was then run through the semi preparative HPLC system. Fractions were collected at the retention times that corresponded to the retention times of the authentic, unlabeled L-tagatose standard. These fractions were then lyophilized and weighed out. The lyophilized 5-13C-L-tagatose product was subjected to 13C NMR.


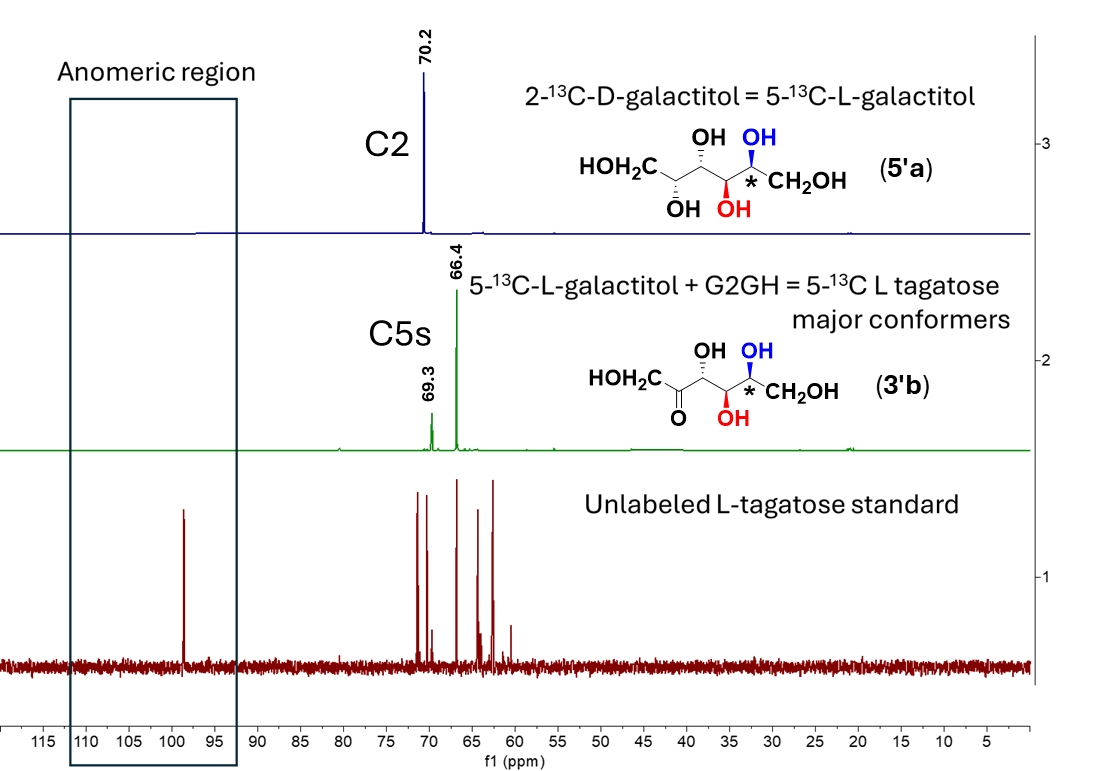


**Fig S7:** Purification protocol of 5-^13^C-L-tagatose and Proton decoupled ^13^C NMR spectra of 2-13C-D-galactitol (δ 70.2 ppm, ^13^C2top), Product 5-^13^C-L-tagatose (δ 66.4 and δ 69.3 ppm, ^13^C5 of conformers, middle) and unlabeled L-tagatose (bottom panel). ^13^C2 did not convert to anomeric carbon supports the production of L-tagatose confirmed by GC/MS analysis.
